# Parents’ understanding and attitudes toward the use of the WeChat platform for postoperative follow-up management of children with congenital heart disease

**DOI:** 10.1186/s13019-023-02153-0

**Published:** 2023-02-08

**Authors:** Wen-Hao Lin, Yu-Kun Chen, Shi-Hao Lin, Hua Cao, Qiang Chen

**Affiliations:** Department of Cardiac Surgery, Fujian Children’s Hospital (Fujian Branch of Shanghai Children’s Medical Center), College of Clinical Medicine for Obstetrics & Gynecology and Pediatrics, Fujian Medical University, Fuzhou, China

**Keywords:** Understanding, Attitude, WeChat, Follow-up, Congenital heart disease, Post-operation

## Abstract

**Objective:**

This study aimed to investigate parents’ understanding and attitudes toward using the WeChat platform for postoperative follow-up management of children with congenital heart disease (CHD).

**Methods:**

A total of 196 children with CHD were followed up using the WeChat platform. A self-designed questionnaire was administered to their parents at discharge.

**Results:**

Only 188 parents completed the questionnaire. One hundred nineteen parents (63.3%) confirmed that they heard about using the WeChat platform for postoperative follow-up, and 104 (87.4%) of them expressed that they were willing to accept the WeChat platform for their follow-up. A total of 42 parents (35.3%) were willing to undergo a follow-up of 1 to 3 months, and 32 (26.8%) were willing to undergo a long-term follow-up. Eighty parents (67.2%) had a positive attitude toward the effect of the WeChat platform on follow-up. Parents in rural areas and those educated at the middle school level or below were more willing to engage with the WeChat platform for postoperative follow-up management (P < 0.05).

**Conclusions:**

Although the WeChat platform is an essential tool for daily communication, its application in postoperative follow-up management is still under study. Most parents who participated in the follow-up study had a positive attitude toward the WeChat platform, especially those in rural areas and with lower education levels.

## Introduction

Congenital heart disease (CHD) is a severe congenital structural malformation, and the health condition of children with CHD is life-threatening and unpredictable [1, 2]. Complications such as pulmonary infection, malnutrition, cardiac insufficiency, and arrhythmia are easy to occur after cardiac surgery [3]. Therefore, close follow-up and professional and meticulous care are still needed for these patients after discharge to reduce complications. However, under the current medical model, parents receive medical support and care guidance during hospitalization, which is terminated once the patient is discharged from the hospital [4]. Due to the lack of professional knowledge and nursing skills, parents face challenges in early care after discharge, which puts tremendous pressure and burden on them [5, 6]. Therefore, it is imperative to carry out effective follow-up management for them.

The WeChat platform is a simple, user-friendly, cost-effective, multi-purpose communication application that consumes fewer resources [7]. The application of WeChat can give patients more real-time and efficient health education and also improve the medication compliance of patients, which have a positive effect on postoperative recovery [8, 9]. The remote follow-up using the WeChat platform can significantly reduce patients’ consultation time and eliminate unnecessary travel. Especially during the COVID-19 pandemic, remote follow-up has its unique advantages [10–12]. Evaluation of parents’ understanding and attitude towards using WeChat for postoperative follow-up management is helpful to carry out remote follow-up better and more effectively via the WeChat platform. However, studies have yet to be conducted. This study investigated parents’ understanding and attitudes toward using the WeChat platform for postoperative follow-up management of patients with CHD.

## Materials and methods

This study included all parents of children after CHD surgery in our center from January 2020 to June 2021. To our knowledge, there was no previous evidence on this topic. Thus, we conducted this study as exploratory research and included all the patients after CHD surgery in our center during the study period to investigate this question. Inclusion criteria of participants: the parents of children younger than one-year-old who received their first cardiac surgery in our hospital from January 2020 to June 2021. Exclusion criteria: (1) Children who were complicated with other diseases. (2) Children with severe postoperative complications or death. (3) Parents could not use the WeChat platform or could not complete the questionnaire. (4) Parents who were unwilling to sign the informed consent form and participate in this study. Our hospital is a provincial children’s hospital. The cardiac surgery department is a mature pediatric heart center with an excellent surgical team and advanced medical equipment. Our team has undergone pediatric cardiac surgery for many years and can complete all CHD operations. A total of 196 parents of children with CHD participated in the study, and 188 participants completed the questionnaire.

The traditional follow-up method was stated as follows: at discharge time, the doctor explained the precautions after discharge, including rehabilitation guidance, diet, feeding, ways of taking medicine, etc. A health education manual containing the precautions after release was distributed to parents. The patients were followed up and received echocardiography at one month, three months, six months, one year, and then every year after discharge.

In the WeChat follow-up method, all parents were required to attend a training program before discharge; the training program included instructions on using the WeChat platform and sending and receiving information in various formats. Parents were also tested to ensure they could proficiently use the WeChat platform. All parents added WeChat accounts and were followed up online using the WeChat platform after discharge.

The health education content in the WeChat group included the education module and the question and answer module. 1. The education module included related knowledge on rehabilitation guidance, diet, feeding, methods of taking medicine, etc. 2. Question and answers module: one medical staff was on duty every day and was online in the WeChat group at 18:00–21:00 to address parents’ problems. The medical staff also guided the parents to communicate, discuss, and share the care experience. Echocardiography was performed at one month, three months, six months, one year, and then every year after discharge. After the examination, the report was sent to the doctor through WeChat. After reading the results and asking about the situation of the patients, the doctor gave the corresponding opinion. If there were unclear or severe postoperative problems, we would notify parents to come to the outpatient for further review via WeChat.

At discharge, parents were asked to complete a questionnaire designed by the researchers. Both parents participated but were taken as a single response. The data were collected from three aspects: (1) general data of the population (7 items, Table 1), (2) knowledge of postoperative follow-up methods and use of the WeChat platform (4 items), and (3) attitude toward the application of the WeChat platform for postoperative follow-up (7 items). Each single-choice question had 2 to 5 answer choices. If the participants needed clarification about any questions, researchers could explain or translate the questions into the participant's local language. After completing the questionnaire, other researchers collected and analyzed the data separately. All questions in the questionnaire and related results are shown in the tables.

**Table 1:** Baseline sociodemographic characteristics of participants

Characteristics	N = 188(n%)
Average duration of hospital stay (day)	13.5
Age range (years)	
20–25	90 (47.9)
26–30	56 (29.8)
31–35	32 (17.0)
> 35	10 (5.3)
Marriage	
Married	175 (93.1)
Divorced	13 (6.9)
Number of children	
One	149 (79.2)
Two	38 (20.2)
Three and more	1 (0.6)
Education	
Junior high school or below	24 (12.8)
Senior high school	68 (36.1)
Bachelor degree or above	96 (51.1)
Career	
White-collar workers and civil servants	58 (30.8)
Professional person	55 (29.3)
Freelancer	34 (18.1)
Other	41 (21.8)
Residential	
City	77 (40.9)
Rural	36 (19.1)
Rural–urban continuum	75 (36.0)
Monthly income (US dollar)	
< 1000	20 (10.6)
1001–2000	75 (39.9)
2001–3000	53 (28.1)
3001–4000	34 (18.1)
> 4000	6 (0.3)

Rural–urban continuum: The area between city and rural

Data were analyzed using SPSS 25.0. Descriptive statistics were used to determine the demographic characteristics of the participants and their knowledge and attitudes toward the application of the WeChat platform for postoperative follow-up. The chi-squared test was used to compare parents' attitudes toward using the WeChat platform for follow-up. P < 0.05 was defined as statistically significant.

## Results

The participants’ understanding of using WeChat for follow-up and their daily use of the platform is shown in Table 2. Most of the participants knew about telephone follow-up (n = 65, 34.5%) and outpatient follow-up (n = 72, 38.3%), while others heard about WeChat follow-up (n = 24, 12.8%), official account follow-up (n = 11, 5.9%), and app platform follow-up (n = 16, 8.5%). Most of the participants thought a follow-up was necessary (n = 133, 70.7%), 50 parents (26.6%) thought it depended on the situation, and the remaining five parents (2.7%) thought a follow-up was unnecessary. Approximately 95 parents (50.5%) of the participants used WeChat for less than 6 h a day, 85 parents (45.2%) used WeChat for 6–12 h, and eight parents (4.3%) used WeChat for more than 12 h a day. More than half of the participants used WeChat mainly for interpersonal communication (n = 122, 64.9%), 28 parents (14.9%) for “checking moments,” 21 parents (11.2%) for subscription accounts, ten parents (5.3%) for buying and selling goods, and seven parents (3.7%) for other purposes.

**Table 2:** Understanding of postoperative follow-up and the use of WeChat both parents participated but were taken as a single response

Item	N = 188(%)
Q1 Which follow-up method are you most familiar with	
Telephone follow-up	65 (34.5)
Clinical follow-up	72 (38.3)
WeChat follow-up	24 (12.8)
Official Accounts	11 (5.9)
App platform	16 (8.5)
Q2 Do you think postoperative follow-up is necessary?	
Necessary	133 (70.7)
As appropriate	50 (26.6)
Unnecessary	5 (2.7)
Q3 How long you use WeChat every day? (hours)	
0–6	95 (50.5)
6–12	85 (45.2)
> 12	8 (4.3)
Q4 You use WeChat mostly for:	
Chat	122 (64.9)
See the “circle of friends”	28 (14.9)
Look at the subscription number message	21 (11.2)
Buy or sell goods	10 (5.3)
Other	7 (3.7)

Table 3 shows the parents’ attitudes toward using the WeChat platform to follow up on their children with CHD. Only 119 parents (63.3%) of the parents said they had heard of the WeChat platform for postoperative follow-up, while the remaining 69 parents (36.7%) still needed to. Among the participants who attended about using the WeChat platform for follow-up, 104 parents (87.4%) said they were willing to engage with the WeChat platform for follow-up. They thought WeChat was the most crucial method for follow-up (n = 73, 61.3%), while a few thought video communication (n = 18, 15.1%) and the ability to use pictures or videos (n = 19, 16.0%) should be included. Among all the participants, 62 parents (52.1%) believed that the main advantage of using the WeChat platform for follow-up was that the follow-up could be completed without leaving home, and 38 parents (32.0%) believed that more follow-up times were available. Regarding the objective deficiencies of follow-up using the WeChat platform, 56 parents (47.1%) of participants thought that the follow-up doctors could not directly check the vital signs in the children, and 35 parents (29.4%) thought that there were specific requirements for the WeChat operation. The remaining 17 parents (14.3%) were worried about the effect of using the WeChat platform on follow-up. A total of 42 participants (35.3%) underwent continuous follow-up for 1 to 3 months, and 32 parents (26.8%) were willing to undergo long-term follow-up. Most people believed that follow-up using WeChat was effective (n = 80, 67.2%).

**Table 3:** Parents’ attitudes to WeChat follow-up

Item	N = 119	(%)*
Have you heard of WeChat being used in postoperative follow-up?		
Know	119	
Don’t know	21	
Unclear	48	
Which form do you think should WeChat follow-up have		
WeChat group	73	61.3
Video call	18	15.1
Official accounts	4	3.4
Receiving pictures and videos	19	16.0
Other	5	4.2
Are you willing to use WeChat for follow-up		
Willing	104	87.4
Uncertainty	13	10.9
Unwilling	2	1.7
What do you think are the most significant benefits of WeChat follow-up		
Complete the follow-up at home	62	52.1
Save on transportation and accommodation	8	6.7
More intuitive information like pictures and videos	11	9.2
Follow-up time is more free	38	32.0
What do you think WeChat follow-up is insufficient		
Certain requirements for WeChat operation	35	29.4
Weak communication signal affects follow-up quality	11	9.2
Doctors can’t directly check the children’s vital signs	56	47.1
Concerning about the effect of WeChat follow-up	17	14.3
Do you think wechat follow-up is effective		
Effective	80	67.2
Futile	32	26.9
Unclear	7	5.9
The duration of WeChat follow-up that you can accept is		
Within a week	4	3.4
A week to a month	27	22.7
A month to three months	42	35.3
Three months to six months	14	11.8
Long-term follow-up	32	26.8

N: the number of participants who had heard of WeChat follow-up. *: Accounted for the percentage of the total number of follow-up visits on WeChat

We analyzed the reflect factors associated with parents’ attitudes to WeChat. Table 4 shows that parents whose families live in rural areas are more willing to accept WeChat platform follow-up (P = 0.014). In addition, parents who were educated at the middle school level or below also had higher acceptance of WeChat platform follow-up (P = 0.006). Parents of patients with complex CHD were more willing to accept WeChat platform follow-up (P = 0.024), and younger parents were more willing to take WeChat platform follow-up (P = 0.011). Parents’ attitudes to WeChat follow-up were not associated with income.

**Table 4:** Reflect factors associated with parents’ attitude to WeChat follow-up

	Willing to accept WeChat follow-up	Unwilling to accept WeChat follow-up and Uncertainty	X^2^	*P*
Residential				
City and Rural–urban continuum	110	42	5.954	0.014
Rural	33	3		
Education				
Middle school and below	78	14	7.522	0.006
Bachelor’s Degree or above	65	31		
Age of parents (years)				
20–25	72	18	10.961	0.011
26–30	46	10		
31–35	21	11		
> 35	4	6		
Monthly income (US dollar)				
< 1000	14	6	3.526	0.473
1001–2000	61	14		
2001–3000	42	12		
3001–4000	22	11		
> 4000	4	2		
The type of CHD				
Simple CHD	117	43	5.0961	0.024
Complex CHD	26	2		

Simple CHD including: ventricular septal defects, atrial septal defects, patent ductus arteriosus. Complex CHD including: tetralogy of Fallot, total anomalous pulmonary vein connection, aortic coarctation

## Discussion

CHD is more complex than other congenital malformations. Postoperative complications are prone to occur, and the care of CHD patients is challenging [3]. Postoperative follow-up is essential for the parents of children with CHD [12–14]. Effective postoperative follow-up can promptly identify the occurrence of postoperative complications and take measures to address them on time. In addition, follow-up can determine whether parents consider caring for their children a psychological burden and help parents cope with these problems correctly. We found that follow-up management typically focused on detecting recurrent disease, monitoring treatment outcomes, and providing ongoing support to patients and their families. Good evidence supported that patients and their families highly valued follow-up care management [15].

As technology advances, follow-up methods are constantly evolving. Each approach has its pros and cons, from the most traditional way of outpatient follow-up to using email, telephone, and mobile medical technology for follow-up [16]. Mobile medical technology has been widely used as an educational tool in healthcare services [17, 18]. In Europe, mobile apps have been used to promote health education for older people, maintain antiviral therapy, and reduce anxiety levels in HIV patients [19]. In the U.K., mobile apps were used to encourage cancer survivors to participate actively in physical health programs [20]. Mobile apps were also used for preoperative preparation before colonoscopy [21]. Presently, WeChat is the most widely used mobile application in China. The skill of using the WeChat platform for daily online communication has become essential for Chinese people due to its simple and convenient operation. Among the parents who completed the questionnaire, 48.5% used the WeChat platform to chat for more than 6 h a day. Most parents had a positive attitude toward using the WeChat platform to manage their patient’s postoperative care and believed that using the WeChat platform for follow-up should be encouraged and used to improve the convenience and efficiency of doctor-patient communication. Parents could receive relevant care education and provide more timely feedback on the patient’s situation to address the nursing requirements at different periods.

Evaluation of parents’ understanding and attitude towards using WeChat for postoperative follow-up management is helpful to carry out remote follow-up better and more effectively via the WeChat platform. This study initially investigated parents’ understanding and attitudes toward using the WeChat platform for postoperative follow-up management of patients with CHD. Our research showed 104 parents (87.4%) were willing to use WeChat for follow-up so that most parents could accept the WeChat platform for postoperative follow-up management. Studies have shown that using the WeChat platform to carry out health care education for perioperative and postoperative family members of children could significantly improve their understanding of the disease and postoperative care level and reduce their anxiety level [22–24]. Parents could quickly and easily obtain the information they needed through the WeChat platform and gradually understood the measures and importance of cardiac rehabilitation. Our study found that parents with lower education had a higher acceptance of the WeChat platform for follow-up management, which might be because their access to health information and nursing knowledge was less than that of parents with higher education. But they could also master the health information and rehabilitation measures provided by the WeChat platform. Parents with higher education levels might be more inclined to acquire relevant nursing knowledge by themselves and have a more comprehensive range of knowledge sources than parents with lower education levels.

Compared with simple CHD, parents of patients with complex CHD would face more significant home care difficulties, their care burden was heavier, and their anxiety was more serious. And repeated follow-up could also reduce their anxiety and depression. First of all, doctors timely solved parents’ doubts through the WeChat platform and corrected wrong ideas to improve cardiac rehabilitation's standardization and effectiveness, which could save the parents’ time and economic costs [25]. Secondly, we could establish a “WeChat group” to enable doctors and patients’ families to discuss and communicate about their medical conditions anytime and anywhere, share their own experiences and rehabilitation results, and maximize effective information exchange and advantages of WeChat platform follow-up management. The visual video function of the WeChat platform was more likely to increase parents’ trust in medical staff than in pure language communication. The diversity of information-sharing formats on the WeChat platform ensured the accuracy and professionalism of follow-up education. It made it easier for patients’ families to understand and accept doctors’ advice [26, 27]. Our study found that parents of patients with complex CHD had a higher acceptance of the WeChat platform for follow-up management. They could obtain high-quality medical support via WeChat follow-up management.

Although transportation in China has become very convenient, routine outpatient follow-up visits are time-consuming and require understanding for parents who live in relatively distant districts. Currently, timely and efficient use of the WeChat platform for follow-up reduces the travel-related burden on parents, eliminates unnecessary trouble, and effectively reduces the rate of loss to follow-up. During the global epidemic, frequent commutes may increase the risk of viral infection. Using the WeChat platform for the follow-up allows parents to complete the follow-up visit in their own homes. At the same time, due to hospitals’ strict policies to prevent the transmission of COVID-19, the need to visit the hospital is eliminated, and the follow-up experience is unaffected. Our study confirmed that parents living in distant rural areas had a more positive attitude toward the WeChat platform for follow-up management. Because young people were generally more impatient and receptive to new things, and outpatient follow-up procedures were cumbersome, our study also found that younger parents preferred WeChat follow-up.

WeChat follow-up management has many advantages, but there are also some shortcomings. First, WeChat follow-up requires equipment and the Internet, and weak communication signals would affect follow-up quality. Therefore, WeChat follow-up was only suitable for some and everywhere. Second, doctors can’t directly check the patient’s vital signs. This situation is the most common concern of parents and the most obvious shortcoming. Third, as a new approach to follow-up, parents are usually worried about whether this approach is advanced and ideal. Therefore, we suggest that WeChat follow-up can be used as an essential supplement for outpatient follow-up. When the examination is normal, WeChat follow-up is enough to complete the follow-up process. When the patient is abnormal, we can also call the parents to the outpatient for review through WeChat. In an emergency, we can also give medical advice through WeChat before arriving at the hospital.

## Limitations

This study had some limitations: (1) This was a cross-sectional analysis with relatively small sample size. Our previous research results confirmed that using WeChat positively affected postoperative follow-up of children with CHD [3, 25, 28], and this study reported parents’ understanding and attitudes toward using WeChat in postoperative follow-up. Therefore, no change in patient outcome was included in this study. (2) This type of follow-up method appeals to specific geographic regions, and whether it can be popularized and applied deserves further study. (3) There was no standard to measure the effectiveness of follow-up. (4) Since this study was exploratory, we did not calculate the sample size and did not evaluate the reliability and validity of the scale.

## Conclusion

Although the WeChat platform is an essential tool for daily communication, its application in postoperative follow-up management is still under study. Parents of patients with complex CHD from rural areas, with low education levels and young ages, were more willing to accept WeChat follow-up.

## Data Availability

The datasets used and analyzed during the current study are available from the corresponding author at the reasonable request.
